# Electrochemical Performance of Citric Acid-Modified α-MnO_2_ Cathode for Aqueous Zinc-Ion Batteries

**DOI:** 10.3390/ijms27219857

**Published:** 2026-11-03

**Authors:** Huan Yu, Yutong Jia, Mingting Liu, Man Li, Xianghua Zhang, Weiwei Han

**Affiliations:** 1 Hubei Key Laboratory of Low-Carbon and Safe Storage & Transportation of Oil, Gas and New Energy, Yangtze University, Wuhan 430100, China; 2 School of Petroleum Engineering, Yangtze University, Wuhan 430100, China; 3 State Key Laboratory of Low Carbon Catalysis and Carbon Dioxide Utilization, Yangtze University, Wuhan 430100, China; 4 Department of Physics and Semiconductor Science, Gachon University, Seongnam-si 13120, Gyeonggi-do, Republic of Korea; 5 Shandong Provincial Key Laboratory of Chemical Energy Storage and Novel Cell Technology, School of Energy Science and Technology, Liaocheng University, Liaocheng 252059, China

**Keywords:** aqueous zinc-ion battery, α-MnO_2_, citric acid modification, cathode material, cycling stability, electrochemical performance

## Abstract

Aqueous zinc-ion batteries (AZIBs) are considered promising for large-scale energy storage owing to their intrinsic safety, low cost, and environmental friendliness. α-MnO_2_, with its unique 2 × 2 tunnel structure, is regarded as one of the most promising cathode materials for zinc-ion batteries; however, its cycling stability is poor due to the disproportionation and dissolution of Mn^3+^ and structural collapse. In this work, α-MnO_2_ was prepared by a hydrothermal method with citric acid introduced into the precursor solution, yielding a MnO_2_@CA cathode material. Results show that the citric acid modification does not change the main crystal structure of α-MnO_2_, and SEM images reveal that both the pristine and modified samples present nanorod morphology. Electrochemical tests demonstrate that after 100 cycles at 0.5 A·g^−1^, the MnO_2_@CA electrode still retains a discharge-specific capacity of about 200 mAh·g^−1^ with a capacity retention of about 90%, while the pristine α-MnO_2_ electrode decays to about 75 mAh·g^−1^. At 0.1 and 0.2 A·g^−1^, MnO_2_@CA delivers higher specific capacities than pristine α-MnO_2_, and it also shows better capacity recovery after the current density returns to 0.1 A·g^−1^. At high rates, the rate performance is somewhat limited by solid-state diffusion/concentration polarization after low-rate activation, while the long-term cycling stability is significantly improved. Kinetics analysis indicates that the electrode process is dominated by diffusion control, while the capacitive contribution increases monotonically with scan rate (from about 7% to 19%) but remains minor compared with the diffusion contribution. Citric acid modification is proposed to stabilize Mn species through chelation, thereby suppressing Mn dissolution, and alleviating structural stress, thereby effectively enhancing the cycling stability and low-rate discharge capacity of the α-MnO_2_ cathode.

## 1. Introduction

Global energy crisis and environmental pollution are becoming increasingly serious, driving the rapid development of new energy technologies. Secondary batteries have become a research hotspot due to their clean and efficient energy storage characteristics [1,2,3,4,5,6,7]. Among them, although lithium-ion batteries are technologically mature and have high energy density, they are limited by the scarcity of lithium resources, and their organic electrolytes are flammable and explosive, posing safety hazards and environmental pollution problems [8]. In contrast, aqueous zinc-ion batteries use neutral or weakly acidic aqueous electrolytes instead of flammable organic electrolytes, and have the advantages of intrinsic safety, low cost, high ionic conductivity, and environmental friendliness, showing broad application prospects in the field of large-scale energy storage [2,3,5,9]. Zinc is abundant in the Earth’s crust, low-cost, non-toxic, and easy to recycle. The zinc anode has a theoretical specific capacity as high as 820 mAh·g^−1^, a relatively low standard electrode potential (−0.762 V vs. SHE), and a high hydrogen evolution overpotential, which are conducive to the uniform deposition and stripping of zinc, endowing aqueous zinc-ion batteries with both high energy density and long-cycle potential [10]. In addition, from the perspective of full life-cycle sustainability, aqueous zinc-ion batteries also have unique advantages in terms of material resources, electrolyte environmental compatibility, and recycling [5,11,12,13,14].

Among various cathode materials, manganese-based oxides have attracted much attention due to their abundant resources, low price, low toxicity, and relatively high operating voltage [2,15]. Among them, MnO_2_ has various crystal forms such as α, β, γ, δ, and λ, and its energy storage behavior is closely related to its crystal structure [15]. α-MnO_2_ has a 2 × 2 tunnel structure (tunnel size of about 0.46 nm) constructed by double chains of [MnO_6_] octahedra, which is favorable for the insertion and extraction of Zn^2+^, and has a theoretical specific capacity of about 308 mAh·g^−1^, so it is regarded as one of the most promising cathode materials for zinc-ion batteries [15]. However, the reaction mechanism of MnO_2_ cathodes is still controversial to date. Pan et al. [16] revealed the reversible chemical conversion reaction mechanism between α-MnO_2_ and H^+^ (MnO_2_ + H^+^ + e^−^ → MnOOH) in aqueous Zn/MnO_2_ batteries by means of ex-situ XRD, TEM, and other characterization methods, and found that pre-adding Mn^2+^ to the electrolyte can effectively suppress Mn dissolution, enabling the battery to maintain a capacity retention as high as 92% after 5000 cycles at a 5C rate. Sun et al. [17] further proposed a stepwise co-insertion energy storage mechanism of H^+^ and Zn^2+^: during discharge, H^+^ is first inserted to form MnOOH, and then Zn^2+^ is inserted to form ZnMn_2_O_4_, accompanied by the reversible formation and decomposition of zinc hydroxide sulfate (ZHS) by-products [18]. In recent years, studies based on controlled experiments in large-volume beaker cells [19], nanoscale STEM-EELS valence-state quantification [20], operando 1H NMR [21], and first-principles calculations [22] have further indicated that the Mn^2+^ to MnO_2_ dissolution-deposition process and the reversible ZHS chalcophanite phase transition during cycling may play a dominant role in the capacity contribution. It can be seen that the zinc storage mechanism of α-MnO_2_ involves the coexistence of multiple processes such as insertion, conversion, and dissolution-deposition, and the dominant mechanism varies with the crystal form, morphology, and electrolyte conditions, which still needs to be determined in combination with specific systems [15,19,20,21,22].

Meanwhile, MnO_2_ cathodes still face problems such as Mn dissolution loss caused by the disproportionation reaction of Mn^3+^, structural collapse caused by Jahn-Teller distortion, low intrinsic electronic conductivity, and sluggish diffusion kinetics arising from the high charge density of Zn^2+^, which severely restrict their cycling stability and rate performance [15,23]. To this end, researchers have developed a variety of modification strategies: compositing with conductive carbon materials to improve electronic conductivity and buffer volume changes [24]; oxygen vacancy/defect engineering to enhance Zn^2+^ adsorption and surface capacitive contribution [25,26]; constructing amorphous nanosheets rich in oxygen vacancies to promote the rapid diffusion of H^+^/Zn^2+^ and stabilize Mn^3+^ [27]; regulating crystal forms and multiphase structures to improve insertion kinetics [28]; and tailoring the morphology and crystal form of nano-MnO_2_ through synthesis methods and surface modification [29]. In addition, electrolytic Zn-MnO_2_ systems can achieve a theoretical capacity of about 616 mAh·g^−1^ through the two-electron Mn^2+^/MnO_2_ deposition–dissolution process, providing a new pathway for improving the energy density of manganese-based cathodes [30]. Complementary interphase and electrolyte strategies have also been developed to regulate Zn/MnO_2_ reaction reversibility and interfacial stability. For instance, the in situ formation of a liquid-crystal interphase in electrolytes with soft templating effects has been shown to improve the reversibility of aqueous dual-electrode-free batteries [31], and upcycling nanoscale fragments from industrial waste liquids has provided sustainable electrolytes for aqueous batteries [32]. Unlike these approaches that modify the electrolyte environment, the present work focuses on direct surface chelation of citric acid on the MnO_2_ cathode to suppress Mn dissolution and stabilize the tunnel structure.

In terms of organic molecular modification, citric acid is a tricarboxylic acid with strong chelating ability, and its carboxyl groups can coordinate with Mn^3+^/Mn^2+^, offering the possibility of surface modification of the MnO_2_ that suppresses Mn dissolution and stabilizes the tunnel structure. Based on this, in this work, α-MnO_2_ was synthesized by a hydrothermal method with citric acid added to the precursor solution to prepare a citric acid-modified α-MnO_2_ (MnO_2_@CA) cathode material. The phase structure, micromorphology, cycling performance, rate performance, and reaction kinetics of the material were systematically investigated by means of XRD, SEM, CV, galvanostatic charge–discharge, and EIS, and compared with those of pristine α-MnO_2_ to explore the mechanism by which citric acid modification improves the electrochemical performance of the electrode.

## 2. Results and Discussion

**Figure 1:**
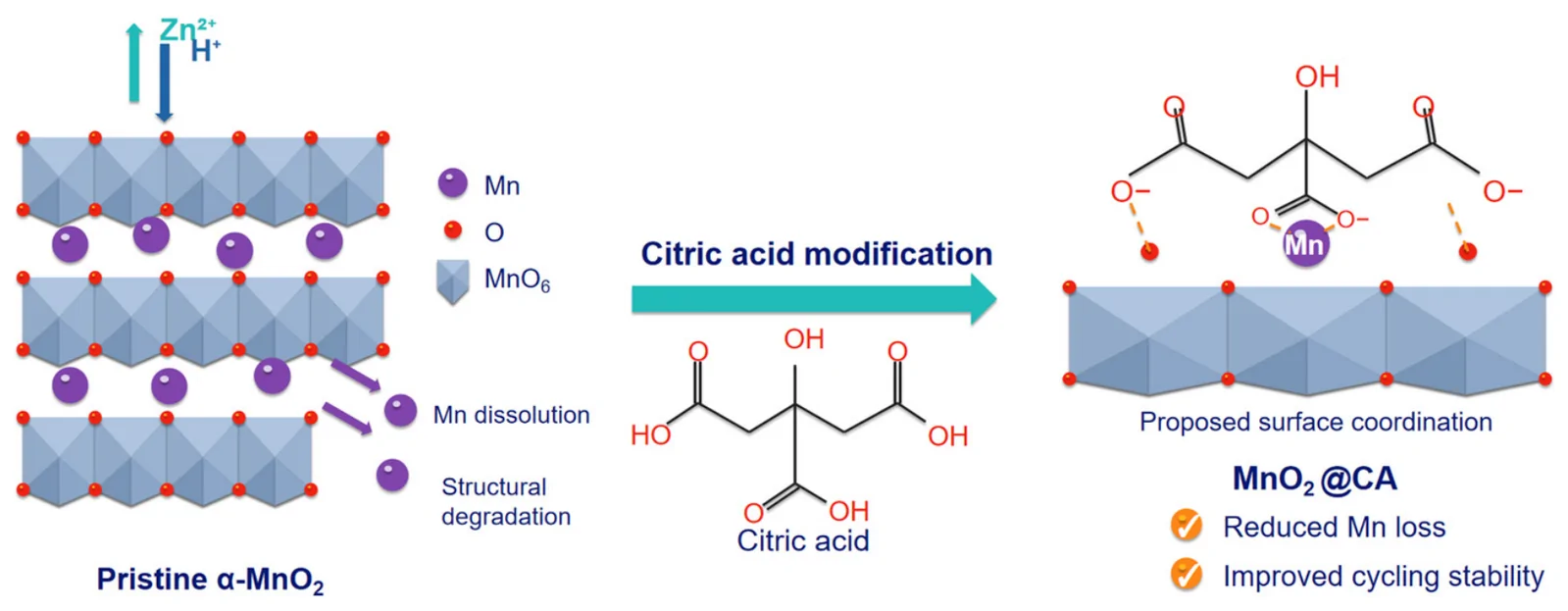
Schematic illustration of the structural degradation of pristine α-MnO_2_ and the proposed stabilization mechanism via citric acid modification. The arrows indicate the insertion/extraction of Zn^2+^/H^+^. The surface modification layer is schematically illustrated; direct observation of the layer is beyond the scope of this work.

The structural degradation mechanism of pristine α-MnO_2_ and the proposed stabilization strategy through citric acid modification are schematically illustrated in Figure 1. Pristine α-MnO_2_ suffers from Mn dissolution and structural degradation during cycling. In contrast, the carboxyl groups of citric acid coordinate with surface Mn species, forming a surface modification that effectively reduces Mn loss and improves the structural stability of the tunnel framework.

**Figure 2:**
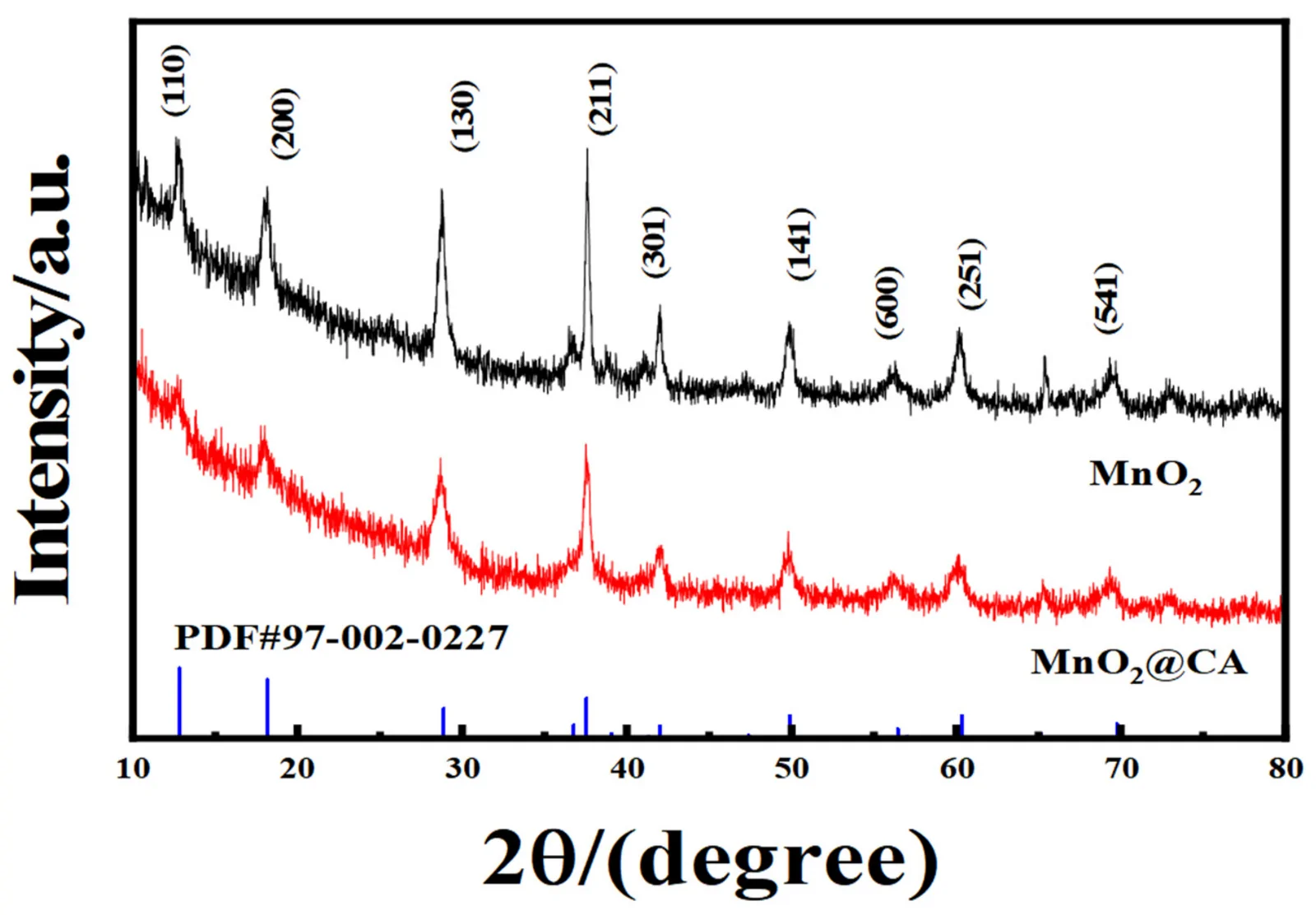
XRD patterns of as-synthesized α-MnO_2_ and MnO_2_@CA.

Figure 2 shows the XRD patterns of α-MnO_2_ and MnO_2_@CA. Both samples exhibit a series of characteristic diffraction peaks in the 2θ range of 20–80°, corresponding to the (110), (200), (130), (211), (301), (141), (600), (251), and (541) crystal planes, respectively, which are consistent with the standard card of α-MnO_2_ (PDF#97-002-0227) [33], indicating that the hydrothermal product is pure-phase α-MnO_2_ without other impurity phases [15]. The stronger (211) reflection than in the calculated PDF card is attributed to the preferred orientation of the hydrothermally grown nanorods, with the (211) planes preferentially exposed. The PDF card intensities are calculated for randomly oriented powders and therefore do not account for the anisotropic nanorod morphology. After citric acid modification, the positions of the diffraction peaks do not shift significantly, indicating that citric acid modification does not change the main crystal form and tunnel structure of α-MnO_2_; however, the overall intensity of the diffraction peaks decreases, which may be attributed to the decreased crystallinity or the smaller grain size after modification.

**Figure 3:**
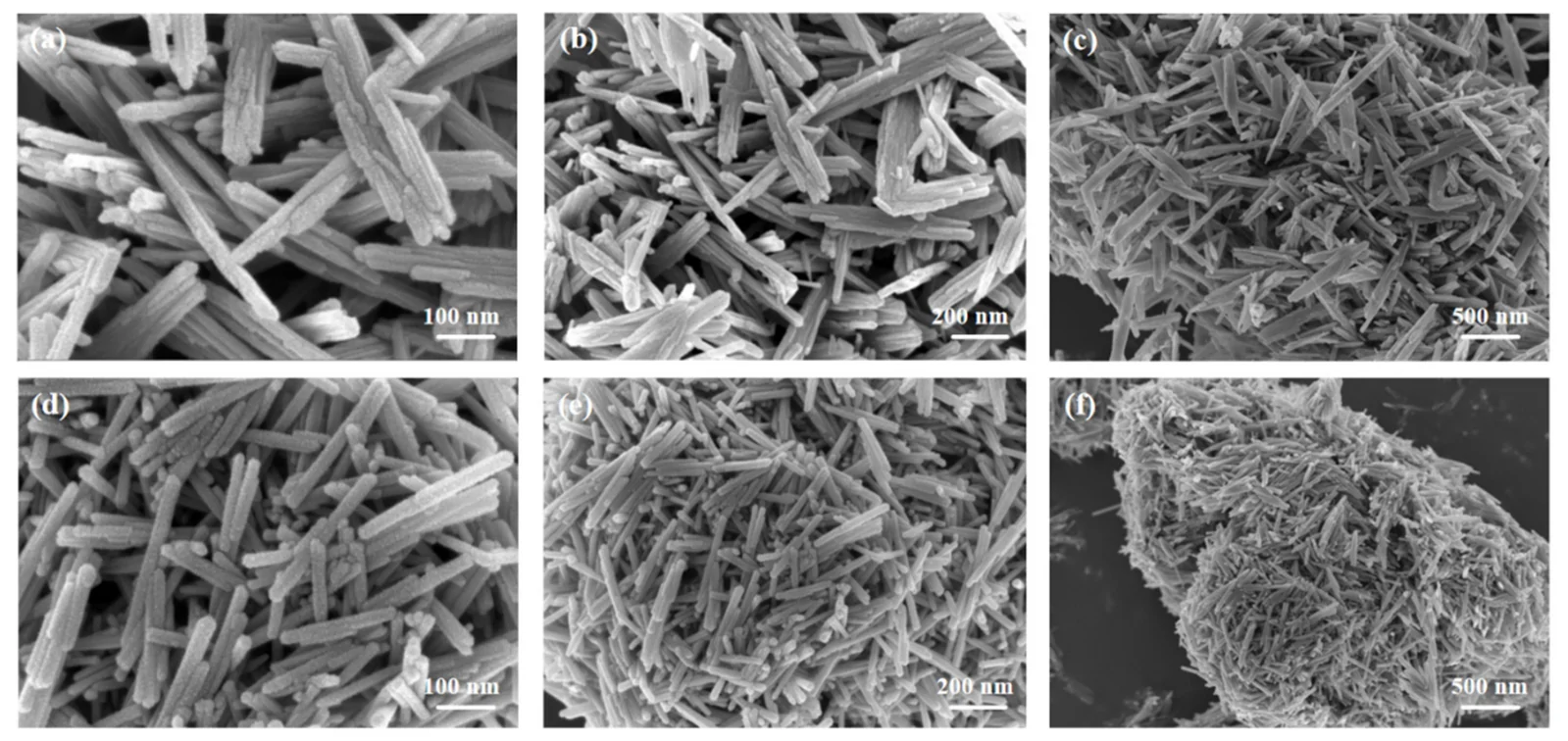
SEM images of (**a**–**c**) α-MnO_2_ and (**d**–**f**) MnO_2_@CA.

Figure 3 shows the SEM images of α-MnO_2_ and MnO_2_@CA. As shown in Figure 3a–c, the hydrothermally synthesized α-MnO_2_ exhibits a one-dimensional nanorod morphology with a relatively smooth surface and a diameter of several tens of nanometers; in Figure 3d–f, MnO_2_@CA still maintains the nanorod morphology with no obvious difference from pristine α-MnO_2_, indicating that citric acid modification does not destroy the nanostructure of α-MnO_2_. The nanorod structure can shorten the solid-state diffusion path of Zn^2+^ and alleviate the volume strain during insertion/extraction, which is beneficial to capacity delivery and structural stability [24].

**Figure 4:**
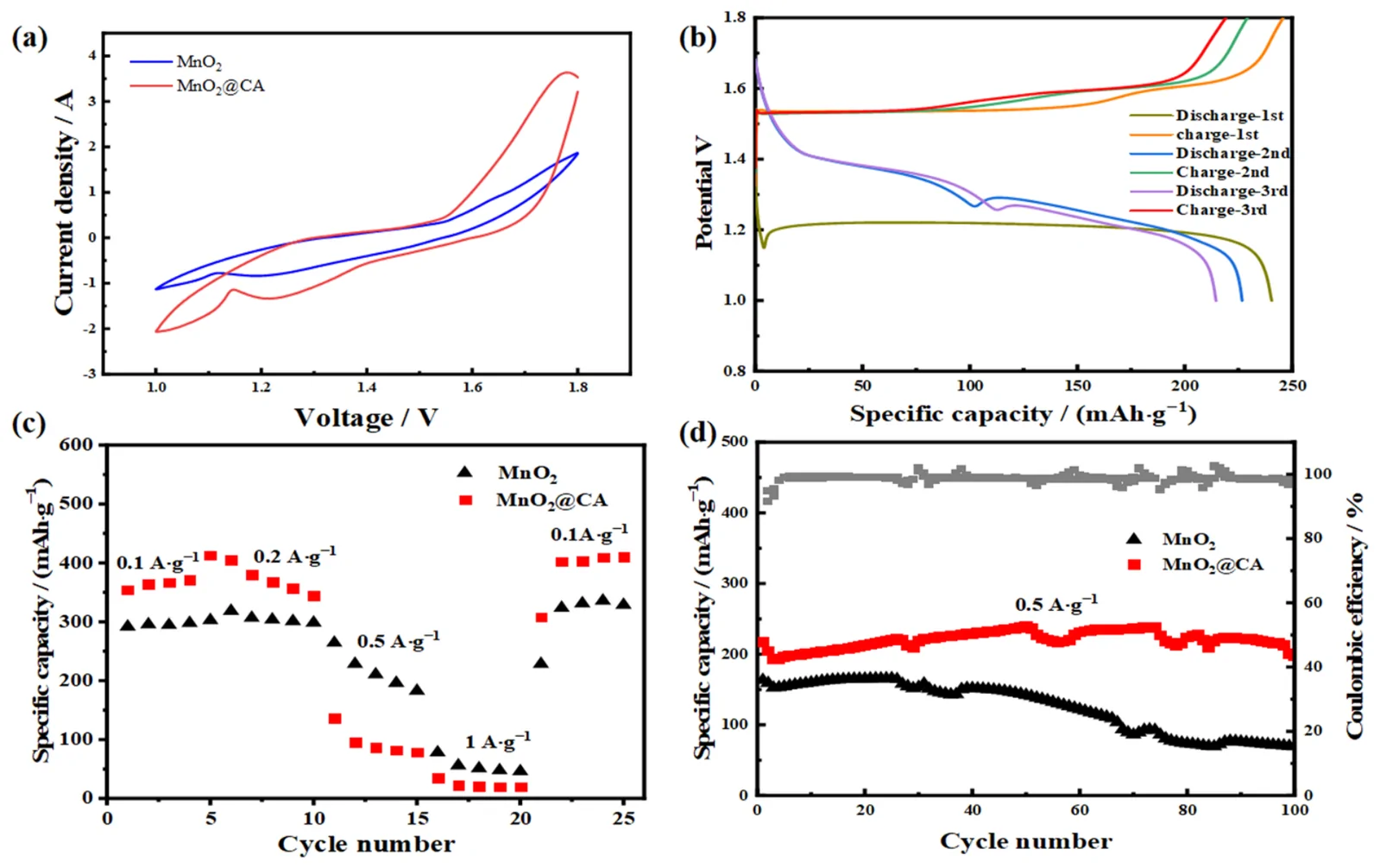
CV curves (**a**), GCD curves (**b**), rate performances (**c**), and cycling performances (**d**) of α-MnO_2_ and MnO_2_@CA.

Figure 4a compares the cyclic voltammetry curves of α-MnO_2_ and MnO_2_@CA electrodes at 2 mV·s^−1^ (1.0–1.8 V). The MnO_2_@CA electrode exhibits clearly defined redox peaks: during discharge, a sharp reduction peak appears at about 1.15 V accompanied by a broader shoulder peak at about 1.3 V, corresponding to the stepwise insertion of H^+^ and Zn^2+^ and the reduction of Mn^4+^ to Mn^3+^/Mn^2+^; during charge, an oxidation peak appears at about 1.75 V, corresponding to the extraction of H^+^/Zn^2+^ and the re-oxidation of Mn^3+^/Mn^2+^ to Mn^4+^, which is consistent with the H^+^/Zn^2+^ co-insertion mechanism reported in the literature [16,17]. Compared with pristine α-MnO_2_, the MnO_2_@CA electrode shows significantly higher redox peak current densities and larger peak areas, indicating higher discharge-specific capacity; meanwhile, the more symmetric peak shapes show that citric acid modification improves reversibility and reduces electrochemical polarization. Figure 4b shows the galvanostatic charge–discharge curves of the electrode in the first three cycles at 0.2 A·g^−1^ (0.8–1.8 V). The initial discharge-specific capacity is about 250 mAh·g^−1^ (first cycle of a fresh cell before activation). The discharge curves exhibit two voltage plateaus at about 1.3 V and 1.2 V, corresponding to the stepwise insertion of H^+^ and Zn^2+^, respectively [16,17], which is consistent with the CV results; the first three cycles basically overlap, indicating good reversibility in the initial cycles.

Figure 4c shows the rate performance of pristine α-MnO_2_ and MnO_2_@CA electrodes at stepwise current densities of 0.1, 0.2, 0.5, and 1 A·g^−1^ (0.8–1.8 V). With increasing current density, the discharge-specific capacities of both electrodes gradually decrease, but the variation trends are significantly different. The similar capacities of MnO_2_@CA at 0.1 and 0.2 A·g^−1^ are attributed to the activation/wetting process and to the more efficient utilization of the reversible Mn^2+^/MnO_2_ deposition–dissolution contribution from the 0.2 M MnSO_4_ electrolyte. At low current densities, MnO_2_@CA exhibits higher discharge-specific capacities: about 390 mAh·g^−1^ at 0.1 A·g^−1^, higher than the approximately 320 mAh·g^−1^ of pristine α-MnO_2_; and it remains at about 345–405 mAh·g^−1^ at 0.2 A·g^−1^, still higher than the approximately 320–340 mAh·g^−1^ of pristine α-MnO_2_. This enhancement is attributed to the citric acid modification, which provides additional active sites at low rates and effectively suppresses Mn dissolution [26,27]. When the current density increases to 0.5 and 1 A·g^−1^, the capacity of MnO_2_@CA decays rapidly and falls below that of pristine α-MnO_2_: it is about 95–155 mAh·g^−1^ at 0.5 A·g^−1^, lower than the approximately 205–285 mAh·g^−1^ of pristine α-MnO_2_, and further drops to about 40–55 mAh·g^−1^ at 1 A·g^−1^, while pristine α-MnO_2_ maintains about 70–100 mAh·g^−1^. This phenomenon is not caused by an increased interfacial charge-transfer resistance, since EIS fitting shows that the Rct of MnO_2_@CA (ca. 139 Ω) is slightly lower than that of pristine MnO_2_ (ca. 141.9 Ω). Instead, the inferior high-rate capacity of MnO_2_@CA is attributed to solid-state diffusion limitation in the bulk and concentration polarization after low-rate activation, which limit the Zn^2+^ insertion/extraction kinetics at high currents. When the current density returns from 1 A·g^−1^ to 0.1 A·g^−1^, the capacity of MnO_2_@CA recovers to about 420 mAh·g^−1^, superior to the approximately 350 mAh·g^−1^ of pristine α-MnO_2_, showing a better capacity recovery capability and confirming that the temporarily low capacity at 0.5 A·g^−1^ resulted from reversible transient polarization rather than irreversible structural damage. The capacity approaching 420 mAh·g^−1^ exceeds the theoretical insertion limit of α-MnO_2_ (ca. 308 mAh·g^−1^), which is attributed to the additional reversible Mn^2+^/MnO_2_ deposition–dissolution contribution from the 0.2 M MnSO_4_ electrolyte and to surface capacitive storage. It should be noted that Figure 4c,d were obtained from different cells under different test protocols and are therefore not directly comparable on a capacity basis. In the rate capability test (Figure 4c), each current step was held for only 5 cycles, so the values at 0.5 and 1 A·g^−1^ represent transient capacities shortly after step switching rather than steady-state values. In contrast, the long-term cycling test (Figure 4d) was conducted at a fixed 0.5 A·g^−1^ from the beginning, allowing the electrode/electrolyte interface and bulk structure to stabilize and reach a capacity of about 200–240 mAh·g^−1^. The difference in the 0.5 A·g^−1^ values therefore originates from the measurement protocol and cell history, not from contradictory material behavior. The data presented are representative; replicate-cell statistics were not available due to the long test duration.

Figure 4d shows the cycling performance of the two electrodes at a fixed current density of 0.5 A·g^−1^ for 100 consecutive cycles, tested on a separate cell from the rate capability test. The initial discharge-specific capacity of the MnO_2_@CA electrode is about 220 mAh·g^−1^, the capacity remains stable in the range of about 200–240 mAh·g^−1^ during cycling, and it still maintains about 200 mAh·g^−1^ after 100 cycles, with a capacity retention of about 90%; the initial discharge-specific capacity of the pristine α-MnO_2_ electrode is about 170 mAh·g^−1^, which decays to about 75 mAh·g^−1^ after 100 cycles, with a capacity retention of about 45%. The coulombic efficiencies of both electrodes are close to 100%. A comparison with representative MnO_2_-based cathodes is summarized in Table S1. Under the conditions listed therein, the MnO_2_@CA electrode delivers a representative capacity of 200 mAh·g^−1^ (peak 220 mAh·g^−1^) with 90% retention after 100 cycles. Although some reported systems show higher retention or capacity, these values were obtained under different electrolytes, current densities, and cycle numbers. The present MnO_2_@CA cathode is therefore notable for its favorable combination of capacity and cycling stability achieved by a one-step citric acid surface modification without additional coating or post-treatment. It can be seen that citric acid modification significantly suppresses the capacity fading during cycling and greatly improves the cycling stability of the α-MnO_2_ cathode. The root cause of the cycling decay of manganese-based cathodes lies in the disproportionation and dissolution of Mn^3+^ and structural collapse [15,23]; the citric acid modification is proposed to suppress the above degradation processes through surface chelation and flexible buffering, thereby significantly improving the long-term cycling stability. In summary, citric acid-modified α-MnO_2_ has a higher discharge-specific capacity and better capacity recovery capability at low rates, but its high-rate performance is somewhat limited by solid-state diffusion in the bulk and concentration polarization after low-rate activation, reflecting the working mechanism of citric acid modification of “sacrificing part of the rate performance in exchange for long-term cycling stability”.

**Figure 5:**
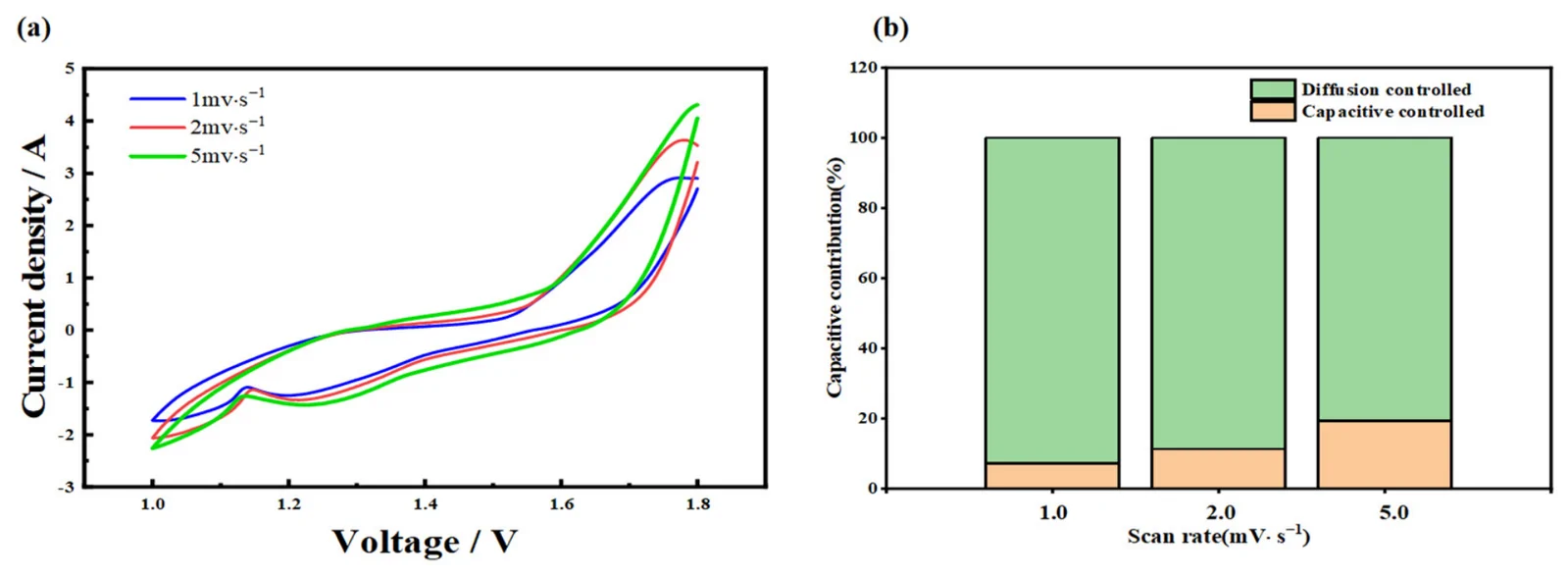
CV curves of MnO_2_@CA at different scan rates (**a**). Capacity contribution rates of MnO_2_@CA (**b**).

**Figure 6:**
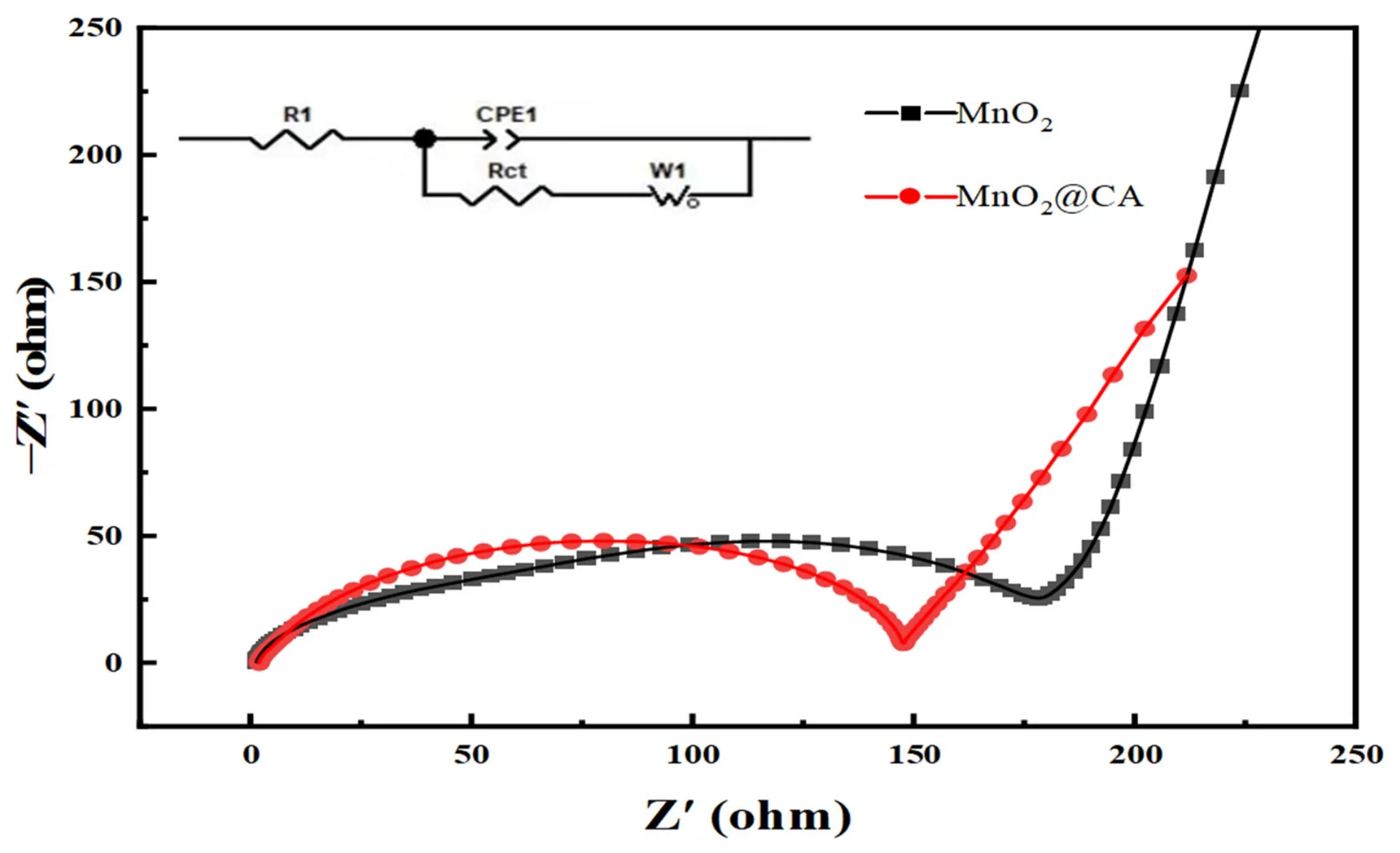
EIS Nyquist plots of α-MnO_2_ and MnO_2_@CA electrodes. The inset shows the equivalent circuit (R1 + Rct//CPE1 + W1) used for fitting.

To further reveal the energy storage kinetics of the MnO_2_@CA electrode, the proportions of capacitive contribution and diffusion-controlled contribution were calculated from the cyclic voltammetry curves at different scan rates, and the results are shown in Figure 5. Figure 5a shows the cyclic voltammetry curves of the MnO_2_@CA electrode at scan rates of 1, 2, and 5 mV·s^−1^. With increasing scan rate, the characteristic redox peaks (ca. 1.15 V, 1.3 V, and 1.75 V) remain clearly resolved; the redox peak currents gradually increase, and the peak potential separation slightly widens, indicating that the electrode process is affected by diffusion control and that the polarization increases at high scan rates; however, the curves still retain relatively intact peak shapes, demonstrating that the electrode has a good rate response capability. At scan rates of 1, 2, and 5 mV·s^−1^, the capacitive contributions are only about 7%, 11%, and 19%, respectively (Figure 5b), indicating that the capacity mainly originates from the diffusion-controlled insertion/extraction of Zn^2+^ and H^+^ in the α-MnO_2_ tunnels. The diffusion contribution therefore remains dominant at all scan rates. This indicates that although diffusion control still dominates the overall capacity, the proportion of the surface capacitive contribution increases monotonically with increasing scan rate, which is consistent with the rule that the surface capacitive contribution increases with increasing scan rate in oxygen-deficient MnO_2_ [25,26]. It is worth noting that although the proportion of capacitive contribution increases at high scan rates, the rate capacities of MnO_2_@CA at 0.5 and 1 A·g^−1^ are still lower than those of pristine α-MnO_2_. Combined with the EIS results (see Figure 6), the high-rate performance of MnO_2_@CA is mainly limited by solid-state diffusion in the bulk and by concentration polarization after low-rate activation, rather than by the interfacial charge-transfer resistance. Therefore, citric acid modification improves the cycling stability without increasing the interfacial charge-transfer resistance, although the high-rate capacity still needs to be optimized.

Figure 6 shows the Nyquist plots of α-MnO_2_ and MnO_2_@CA electrodes (0.01 Hz–100 kHz, AC amplitude of 5 mV). Both curves consist of a semicircle in the high-frequency region and a sloping line in the low-frequency region, and have a diameter of about 140 Ω. According to the equivalent circuit fitting (inset of Figure 6), the charge-transfer resistances of pristine α-MnO_2_ and MnO_2_@CA are about 141.9 Ω and 139 Ω, respectively. The semicircle of MnO_2_@CA is slightly smaller, indicating that the citric acid modification layer decreases the interfacial charge-transfer resistance to a certain extent, which is beneficial for interfacial kinetics, consistent with the conclusion that a surface modification can modulate the interfacial ion transport barrier [22]. In the low-frequency region, both electrodes exhibit diffusion slopes, corresponding to the diffusion process of Zn^2+^ in the bulk of the electrode. The low-frequency diffusion characteristics of the two electrodes are similar, indicating that the modification has a relatively limited effect on bulk ion diffusion. Overall, citric acid modification achieves a significant improvement in cycling stability while reducing the interfacial charge-transfer resistance. The high-rate capacity limitation of MnO_2_@CA is therefore attributed to the solid-state diffusion limitation in the bulk or the concentration polarization induced by low-rate activation, rather than the interfacial charge-transfer resistance.

Based on the above results, the improvement of the electrochemical performance of the α-MnO_2_ cathode by citric acid modification can be attributed to the following aspects: (i) Citric acid is a tricarboxylic acid with strong chelating ability, and its surface carboxyl groups can coordinate with Mn^3+^/Mn^2+^, which is proposed to suppress disproportionation reaction of Mn^3+^ and Mn dissolution, which is similar to the mechanism by which Mn^2+^ additives in the electrolyte suppress Mn dissolution [5]; previous studies have shown that oxygen vacancies and amorphous structures can stabilize Mn^3+^ and suppress Jahn–Teller distortion [26,27]. (ii) The surface modification can serve as a flexible buffer layer, alleviating the volume change and structural stress during Zn^2+^ insertion/extraction and maintaining the stability of the tunnel structure [27]. (iii) Polar functional groups such as surface carboxyl groups can improve the wettability between the electrode and the electrolyte and promote ion transport; meanwhile, the surface modification may introduce additional pseudocapacitive active sites, enhancing the surface capacitive contribution [25]. (iv) Although the high-rate capacity of MnO_2_@CA is still limited by solid-state diffusion/concentration polarization, the citric acid modification does not increase the interfacial charge-transfer resistance and is proposed to improve the long-term cycling stability by suppressing Mn dissolution and stabilizing the tunnel structure.

## 3. Experimental Section

### 3.1. Reagents and Instruments

The main reagents and materials used in this experiment include zinc foil, stainless steel foil, glass fiber separator, potassium permanganate (KMnO_4_), manganese sulfate monohydrate (MnSO_4_·H_2_O), acetylene black, polyvinylidene fluoride (PVDF), N-methyl pyrrolidone (NMP), zinc sulfate heptahydrate (ZnSO_4_·7H_2_O), and citric acid. Among them, zinc foil was used as the anode material, stainless steel foil was used as the cathode current collector, and the glass fiber separator was used to isolate the positive and negative electrodes while ensuring electrolyte infiltration and ion transport; KMnO_4_ and MnSO_4_·H_2_O were used for the hydrothermal synthesis of α-MnO_2_, and citric acid was used to modify the MnO_2_ surface during the hydrothermal synthesis; PVDF was used as the binder, NMP was used to disperse the electrode slurry, and acetylene black was used as the conductive agent; ZnSO_4_·7H_2_O and MnSO_4_·H_2_O were used to prepare the aqueous electrolyte containing 2 M ZnSO_4_ + 0.2 M MnSO_4_.

The main instruments used in this experiment include a magnetic stirrer, a vacuum blast drying oven, an electronic balance, an X-ray diffractometer (XRD), a scanning electron microscope (SEM), an electrochemical workstation, a battery testing system, a hydraulic coin cell crimper, and a glove box. XRD was used to analyze the phase structure of the materials, SEM to observe the micromorphology, and the battery testing system and electrochemical workstation for charge–discharge, cycling, rate, CV, and EIS tests.

### 3.2. Preparation of α-MnO_2_

α-MnO_2_ was synthesized by a hydrothermal method. In a typical synthesis, MnSO_4_·H_2_O (3 mmol) and H_2_SO_4_ (2 mL) were dissolved in 50 mL of deionized water and stirred for 10 min to form solution A. Meanwhile, KMnO_4_ (2 mmol) was dissolved in 20 mL of deionized water and stirred for 10 min to form solution B. Solution B was then slowly added dropwise to solution A and stirred for another 1 h. The mixture was transferred into a 100 mL Teflon-lined autoclave and heated at 120 °C for 12 h. After natural cooling, the product was collected by vacuum filtration, washed three times with deionized water and anhydrous ethanol, and dried at 80 °C under vacuum to obtain pristine α-MnO_2_.

### 3.3. Preparation of MnO_2_@CA

For MnO_2_@CA, the synthesis was similar to that of pristine α-MnO_2_, except that 87 mg of citric acid was added to the mixed precursor solution under stirring for 15 min before the hydrothermal reaction. The resulting mixture was then transferred into a 100 mL Teflon-lined autoclave and heated at 120 °C for 12 h. Finally, the product was collected, washed three times with deionized water and anhydrous ethanol, and dried at 80 °C to obtain MnO_2_@CA.

### 3.4. Cell Assembly and Electrochemical Measurements

The active material, acetylene black, and PVDF were mixed at a mass ratio of 7:2:1 in NMP. The slurry was coated onto stainless steel foil current collectors, dried under vacuum, and punched into circular electrodes with a diameter of 12 mm. The active-material loading was approximately 1.11 mg cm^−2^, corresponding to about 1.26 mg of active material per electrode (electrode diameter: 12 mm). Using zinc foil as the anode, glass fiber filter paper as the separator, and 200 μL of 2 M ZnSO_4_ + 0.2 M MnSO_4_ aqueous solution as the electrolyte (Mn^2+^ was pre-added to suppress Mn dissolution of the cathode [16]), CR2032 coin cells were assembled in a glove box. Specific capacities were normalized by the mass of active material, which was 1.26 mg per electrode. The voltage window for CV tests was 1.0–1.8 V with scan rates of 1, 2, and 5 mV·s^−1^; the voltage window for galvanostatic charge–discharge tests was 0.8–1.8 V with current densities of 0.1–1 A·g^−1^. In the rate capability test, each current density step (0.1, 0.2, 0.5, and 1 A·g^−1^) was maintained for 5 cycles before switching to the next step. The rate capability test and the long-term cycling test were performed on separate cells. The lower cutoff of 0.8 V for GCD allows for complete discharge to maximize capacity utilization, while CV measurements were limited to 1.0 V to avoid excessive polarization and structural degradation of the MnO_2_ cathode at high scan rates. The frequency range for EIS tests was 0.01 Hz–100 kHz with an AC amplitude of 5 mV. All electrochemical tests were carried out at room temperature.

## 4. Conclusions

A citric acid-modified α-MnO_2_ cathode (MnO_2_@CA) was prepared by a facile hydrothermal method with in situ citric acid modification. The modified electrode significantly improves the cycling stability of the aqueous zinc-ion battery: after 100 cycles at 0.5 A·g^−1^, it retains a discharge-specific capacity of about 200 mAh·g^−1^ with a capacity retention of about 90%, whereas the pristine α-MnO_2_ electrode decays to about 75 mAh·g^−1^ (ca. 45%). This improvement is proposed to originate from the surface chelation of Mn species by citric acid, which suppresses Mn dissolution and alleviates structural stress, together with improved electrode–electrolyte wettability. It should be noted that direct surface characterizations (such as FTIR, XPS, and ICP measurements of dissolved Mn concentration) were not performed in this preliminary study, and such measurements were not available at the time of this work; the proposed mechanisms therefore remain tentative, and their definitive validation is beyond the scope of the present study.

## Data Availability

The original contributions presented in this study are included in the article/Supplementary Materials. Further inquiries can be directed to the corresponding authors.

## Supplementary Materials

The following supporting information can be downloaded at https://www.mdpi.com/article/10.3390/ijms27219857/s1. References [34,35,36,37,38,39,40] are cited in the Supplementary Materials.
