# Supplementary material for: Electrochemical Performance of Citric Acid-Modified α-MnO2 Cathode for Aqueous Zinc-Ion Batteries
Source: Int J Mol Sci. 2026 Nov 3;27(21):9857. doi: 10.3390/ijms27219857 (PMC13763891; doi:10.3390/ijms27219857)
Supplement: Supplementary file 1 [file ijms-27-09857-s001.zip › ijms-4635040-supplementary.pdf]

Supplementary Materials

Table S1. Comparison of the electrochemical performance of CA-MnO_2 (this work) with previously reported MnO_2-based cathodes for aqueous zinc-ion batteries.

Cathode Material	Capacity (mAh $g^{-1)}$	Current Density (mA $g^{-1)}$	Cycle Number	Capacity Retention	Ref.
CA-MnO_2 (Our work)	215 (Peak 240)	500 (0.5 A $g^{-1)}$	100	89.60%	This work
iPOP@ε-MnO_2(PN-POP)	172.4	0.5 C (rate)	1500 (at 1 C)	74.60%	[1]
Co-coated MnO	192	600	500	61.5% (calc.)	[2]
PANI@MnO_2 (6h)	148	500	500	53.90%	[3]
PPy@MnO_2 (LC/MnO_2/PPy)	128.3	1000	1000 (at 300)	91.40%	[4]
PDA@MnO_2 (Spherical)	186.9 (at 600)	400 ~ 600	500 (at 1000)	33.30%	[5]
SP@Bicrystalline-MnO_2	323.9 (at 600)	400 ~ 600	500	22.30%	[6]
MnO_2 (CS separator)	212	500	1000 (at 1000)	98.40%	[7]

Notes: CA, citric acid; calc., calculated value. Current densities and cycle numbers are presented as reported in the cited references; C-rate values are indicated where applicable. References [1–7] are listed in the Supplementary Materials.

References

1. Chen, S.; Gao, X.; Huang, R.; Fang, S.; Peng, X.; Li, M.; Feng, Y.; Qu, X.; Zhang, X. In situ Composite of Cationic Porous Organic Polymers and ε-MnO2 as Cathode Materials for Aqueous Zinc Ion Batterys. Advanced Functional Materials 2025, 36, doi:10.1002/adfm.202521459.
2. Li, Q.; Zhu, Y.; Wu, S.; Du, W.; Wang, Y.; Feng, S. Co-coating strategy enables robust MnO cathode in aqueous zinc ion batteries. Journal of Energy Storage 2026, 141, doi:10.1016/j.est.2025.119358.
3. Ma, C.; Liu, Y.; Chen, J.; Yang, R.; Du, H.; Yang, Z.; Xu, Y.; Yu, W.; Wang, Y. Flexible aqueous Zn-MnO2 battery with reduced Jahn-Teller effect. Electrochimica Acta 2026, 572, doi:10.1016/j.electacta.2026.149428.
4. Ilyas, S.; Sultana, I.; Kainat, F.; Masood, A.; Iqbal, M.F.; Bano, S.; Ali, W.; Javid, A.; Irfan, I.; Razaq, A. Freestanding and binder free polypyrrole coated MnO2 based bendable electrodes for development of aqueous zinc ion pouch battery cell. Journal of Electroceramics 2026, doi:10.1007/s10832-026-00464-x.
5. Li, S.; Wei, Y.; Wu, Q.; Han, Y.; Qain, G.; Liu, J.; Yang, C. Spherical PDA@MnO2 cathode for rechargeable aqueous zinc ion batteries. Materials Letters 2023, 348, doi:10.1016/j.matlet.2023.134671.
6. Li, S.; Wu, Q.; Gui, D.; Liu, R.; Han, Y.; Cao, X.; Yang, C. SP@Bicrystalline-MnO2 as an advanced cathode for aqueous zinc-ion battery. Materials Letters 2022, 319, doi:10.1016/j.matlet.2022.132288.
7. Yang, X.; Wu, W.; Liu, Y.; Lin, Z.; Sun, X. Chitosan modified filter paper separators with specific ion adsorption to inhibit side reactions and induce uniform Zn deposition for aqueous Zn batteries, Chemical Engineering Journal 2022, 450, 137902, doi:10.1016/j.cej.2022.137902.
